# Clinical Outcome of Medial Opening Wedge Osteotomy with T-Locking Plate : Two Years Follow-Up

**DOI:** 10.5704/MOJ.1403.019

**Published:** 2014-03

**Authors:** W Kongcharoensombat

**Affiliations:** Institute of Orthopaedics, Lerdsin Hospital, Bangkok, Thailand

## Abstract

Objective: This study was undertaken to determine clinical outcome after medial opening wedge osteotomy with Tlocking plate, with two- year follow up. Twenty-two patients (22 knees) who underwent medial opening wedge osteotomy with T-locking plate (stainless steel 316L, 6 holes) for treatment of varus malalignment of the leg between March 2005 and April 2008 were included in the study. The amount of correction ranged from 7° to 19° (mean, 9.77°). Clinical and radiographic findings were evaluated with VAS and the Lysholm score at sixth, twelfth and twenty- fourth months. Follow-up ranged from 18 to 37 months (mean, 2.1 years). Significant reduction was observed of VAS, from 4 (range: 3.5-5) to almost free of symptoms (1.0 to 0.5) at the twentyfourth month follow-up (P<0.01). Good results were achieved in the Lysholm score (P<0.01). Medial opening wedge osteotomy with T-locking plate is safe and efficient procedure for corrective varus deformity of knee.

**Key Words:**

High tibial osteotomy, T-locking plate , medial opening wedge osteotomy, varus deformity

## Introduction

High tibial osteotomy was described by Jackson in 1958 as a means of treating medial unicompartmental arthrosis of the knee joint ^1^. Recently, numerous authors have demonstrated that osteotomies about the knee are an effective treatment modality and has relatively good long-term results ^2^. High tibial osteotomy is indicated for the treatment of symptomatic medial compartment osteoarthritis and is generally reserved for patients up to 60 years of age ^3^. The long-term effect of ligament injuries in active patients leads to progressive deterioration in the articular cartilage of the tibio-femoral joint and the development of medial compartment osteoarthritis ^4^. In patients presenting with ligament-rupture and early arthritis, there is risk of progression of the arthritis with ligament reconstruction procedures ^5^. In genu varus deformity with medial compartment degeneration, high tibial osteotomy (HTO) can stop the progression of arthritis for several years ^6^. The best results following HTO are obtained when it is performed in early arthritis ^7^.

Classically, this procedure has been carried out by closingwedge osteotomies. Recently, advantages have been sited for opening-wedge osteotomies, including ease of procedure and improved accuracy of correction with comparable short-term to midterm results ^8, 9^. Other advantages of medial HTO are preserving the proximal tibial anatomy and bone stock allowing easy conversion later to total knee arthroplasty, avoiding fibular osteotomy, precision in correcting the mechanical axis, and preserving the proximal tibio-fibular joint and peroneal nerve ^10^. However adequate stable fixation is mandatory for sound healing of this additive type of osteotomy in order to minimize the risk of non-union and loss of correction. There are several types of implants for this procedure. The Puddu plate is a short plate comprising an integrated spacer block, available in different sizes. This plate is attached to the medial side of the proximal tibia, with the spacer being inserted into the osteotomy gap, thus blocking closure of the gap and keeping it open. A further implant, specially designed for high tibial opening wedge osteotomy, is a rigid and long titanium plate (Tomofix) which is anatomically precontoured to the medial tibial metaphysis. This implant is equipped with locking bolts and, thus, functions as an internal plate fixator11. Short designs are inferior to longer designs, even if they have angle-stabilized locking screws. In long plates the thickness and rigidity of the material play an important role, with a thin and more flexible plate providing less stability than a thick and rigid plate. Reliable fixation, with sound bone union and longterm maintenance of the correction, can best be achieved with a rigid, long plate fixator with locking bolts.

In particular, the stability of the bone fixation and the ability for bone fusion (union) to occur, as well as maintenance of the achieved correction are probably, important factors with good outcome for the patient ^12^.

In this study, we performed medial opening wedge osteotomy of proximal tibia with T-locking plate (stainless 316L, 6 holes;Synthes) in the patient with varus deformity of the knee.

## Materials and Methods

### 

Twenty-two patients who underwent medial opening wedge osteotomy with T-locking plate [stainless 316L, 6 holes] for treatment of genu varus deformity and pain due to osteoarthritis of medial compartment of the knee or varus deformity causing chronic ligament instability of knee between March 2005 and April 2008 were reviewed in the Institute of Orthopedics, Lerdsin General Hospital, Thailand. Two of the 22 patients were women and twenty patients were men. The mean age was 33.0 years, ranging from 17 to 53 years. The mean follow-up period was twenty five months (range: 18-37 months). Besides the varus deformity in all patients, some also had other knee problems. Five patients had malunion of fracture of tibial plateau, five had anterior cruciate ligament injury, seven had posterior cruciate ligament injury, and two patients had multiple ligament injury (Table I).

**Fig. 1b: The mechanical axis was measured from center of femoral head through center of ankle are shown.**
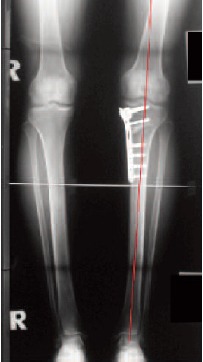


Inclusion criteria: Patients with varus malalignment symptoms with overloading in the medial compartment of the knee from osteoarthritis or knee instability due to chronic ligaments injury were included in this study. The term varus malalignment (Figure 1A) was applied when the tibiofemeoral mechanical axis passed through the medial tibial plateau or when the tibiofemeoral mechanical angle was more than 3 degrees compared to the opposite side. An intact soft-tissue covering of the medial aspect of the proximal tibia and wide lateral joint space were further preconditions for surgery. The range of motion of the knee joint had to be at least 100 degrees from full extension and to flexion.

Exclusion criteria: Patients over the age of 60 years, adolescents with radiological open growth plates or infection of the knee joint were excluded.

Preoperative assessment and planning: Before the surgical procedure, all patients completed subjective administered evaluation forms to obtain Lysholm knee score and the subjective pain intensity was determined by means of a visual analogue scale (VAS) from 0 to 10 (0 = no pain,10 = severe pain) ^13^. The range of passive motion (flexion / extension) was measured with a goniometer. The radiological documentation included standard knee radiographs, a weight-bearing anteroposterior (AP) view, and a lateral view. In patients with suspected additional lesions of the knee joint, magnetic resonance imaging was carried out to plan further operations.

For the preoperative planning, we used the AP weightbearing radiograph. Varus and valgus angulations of the knee14 were by measuring the angle between the femoral and tibial mechanical axes. The mechanical axis of the femur was defined as the line connecting the centres of the hip and the knee joint. The mechanical axis of the tibia was defined as the line connecting the centres of the knee and the ankle joint. Knee alignment (tibio-femoral mechanical angle) was derived by measuring the angle of intersection between these two axes, where 180 degrees equated to a straight line, angles greater than 180 degrees indicated a valgus knee position, and angles less than 180 degrees indicated varus alignment. The weight-bearing line (WBL) was found by drawing a line from the centre of the femoral head to the centre of the ankle mortise (Figure 1A). The horizontal distance from the WBL to the medial edge of the tibial plateau was then divided by the width of the tibial plateau. Thus, a WBL ratio of less than 0.5 indicated varus angulation with the load shifted medially, whereas a value of greater than 0.5 denoted valgus angulation with the load shifted towards the lateral compartment ^15^.

The amount of correction of the mechanical axis was guided by the extent of degenerative changes in the medial joint compartment. If narrowing of the medial joint space was evident on the radiograph, we carried out an overcorrection according to the papers of Fujisawa ^16^(Figure 1A), whereby the weight-bearing line was shifted to a point 62% lateral on the transverse diameter of the tibial plateau. If the overloaded medial compartment was largely intact, without significant narrowing of the joint space, we corrected the axis to neutral with the postoperative weight-bearing line passing the centre of the knee.

Operative technique: The operation was performed under spinal or general anesthesia with the patient in supine position. Intravenous antibiotic was used. A tourniquet cuff was applied on the thigh. The leg was left free of drape, including the iliac crest, so as to be able to check alignment intraoperatively, and under image intensification. A fluoroscope was installed, allowing visualisation of the kneejoint in two planes.

The surgical procedure, was a modified Tomofix-AO surgical technique ^17^. To ensure an intact lateral joint compartment and to treat additional intra-articular lesions, a knee arthroscopy was first performed on every patient. We used an oblique skin incision 4-6cm distal to the joint line, perpendicular to the pes anserinus, for exposure (Figure 2). Proximal to the pes anserinus, the medial collateral ligament was dissected off the posteromedial cortex of the tibia and a blunt Hohmann retractor was inserted to protect the neurovascular structures. The direction of the osteotomy in the coronal plane was marked with a 2.0 mm-threaded Kwire under fluoroscopic control. The osteotomy was started at the upper margin of the pes anserinus and ended 0.5 cm from the lateral cortical margin at the level of the tip of the fibula. The osteotomy was performed in L-shape, in two planes ^17^. The first osteotomy was performed distal to the Kwire, parallel to the tibial slope. The second frontal osteotomy plane started in the anterior one-third of the proximal tibia at an angle of 135 degrees to the first osteotomy plane. This osteotomy exited the bone proximal to the insertion of the patellar tendon. The osteotomies were performed with oscillating saw and were completed with chisels. The osteotomy was opened by stepwise insertion of three chisels to avoid intra-articular fractures of the tibial plateau. The mechanical axis was then adjusted according to the preoperative planning and the correction retained with a bone spreader that was inserted into the posteromedial osteotomy gap. T-locking plate (stainless steel 316L, 6 holes) was inserted into subcutaneous tunnel and centred on the anteromedial plane of the tibia. The proximal fixation of the plate was carried out with three locking head screws in the subcortical area. The plate was then pretensioned by inserting a temporary lag screw distal to the osteotomy. For definitive fixation of the plate, the distal locking head screws were inserted through a small incision (Figure 2). Then the osteotomy gap was closed with bone graft. Finally, the lag screw was replaced by a locking head screw. A suction drain was inserted but placed away from the osteotomy gap.

Postoperative management: Postoperatively, the knee was immobilized for one day with a posterior knee slab. Range of motion exercises and partial weight bearing using crutches were allowed two to three days after surgery and the drain was removed. Full weight bearing was permitted at 5 to 6 weeks post-operation.

Radiological evaluation: The postoperative radiological and clinical follow-up examinations were carried out after six weeks, twelve weeks, six months and twelve months. An AP weight-bearing radiograph was taken on a long cassette at twelve weeks (Figure 1B).

Clinical Assessment: The clinical results were graded with the scale of VAS and the Lysholm score at pre-operation, 6 month, 12 month and 24 month post-operation.

Statistical Analysis: Statistical analyses were performed with SPSS 11.0 for windows (SPSS Inc., Chicago, IL). For statistical evaluation, the nonparametric Mann-Whitney rank sum test was used. P < 0.05 was considered significant. Comparisons were made between scoring (separately for Lysholm and VAS) at 6, 12, and 24 months postoperatively and the preoperative score.

## Results

The Lysholm score was used to compare between preoperative knee function and six months after surgery. Results were achieved. The differentiation is significant (Lysholm: preoperative vs 6 months; P< 0.01). Significant improvement compared to preoperative status was shown by (Lysholm: preoperative vs 12 months, P <0.01) after surgery and after 24 months (Lysholm: preoperative vs 24 months, P <0.01). Finally, a significant increase in function between 12 and 24 months after surgery was observed using Lysholm scores (Lysholm: 12 vs 24 months, P<0.01); (Figure 3).

Another clinical evaluation was determined by using the Visual Analogue Scale (VAS, from 0 no pain to 10 severe pain). The patients had been asked to report the VAS for a significant subjective reduction of pain, from a score of 4 (range: 3.5-5) before the operation and were almost free of pain symptom- (scores of 1.0 to 0.5) under full weightbearing at the follow-up examinations. Walking without crutches and full weight-bearing were achieved after an average of eight weeks (range: 6-10 weeks). A significant decrease of the VAS was detected at 6, 12, and 24 months after surgery (6 and 12m -, P <0.01; 24m -, P< 0.01). However, no difference in the VAS was recorded between 6 and 12 months after surgery (6- 12 month: P=0.745; P >0.05); (Figure 4).

Radiologic Evaluation: Analysis of pre- and postoperative axial alignment revealed an average of 22 mm (range, 11 to 42) medialization of the weight-bearing axis in relation to the anatomic center of the knee on preoperative radiographs. According to the recommendations of various authors 18, 19, 20 a slight overcorrection was performed aiming between 50% and 66% laterally on the transverse diameter of the tibial plateau so that the mean of axial correction was 27.7 mm (range, 20 to 54). The amount of correction ranged from 7° to 19°, with a mean of 9.77°. There were 18 cases with autogenous corticocancellous iliac bone graft and four cases using artificial bone graft. Patients started passive and active motion after the 2nd day of surgery and began partial weight bearing with crutches at 6-8 weeks. Concerning intraoperative complications, an intra-articular fracture of the lateral tibial plateau was observed on a postoperative radiograph, which delayed weight bearing. Ten week later, we found complete- bony union and widening of the medial compartment on radiograph (Figure 5A,B).

All patients were followed-up until bony union of the osteotomy had been radiologically documented. Consolidation of the osteotomy gap was determined (Figure 5C). The average time to bone union was 12.1 weeks (range, 8-16). There was no instance of non-union of the osteotomy gap after the tibial osteotomy. During the whole period of the study, there were no cases of implant failure.

## Discussion

The results of this study shows that medial opening wedge osteotomy with T-locking plate (stainless steel 316L, 6 holes) for corrective varus deformity of knee is good.

In our series of cases, there was a tendency to undercorrect the deformity (mean tibiofemeoral mechanical angle after the operation: 182 degrees) because of the young mean age of the patients (33 years old) and the medial compartment of the knee was in the state of “prearthritis”. None of the patients had genu varus deformity but only ligament injuries of the knees. The effect of a chronic ligament instability due to rupture is an increase in tibia translation, particularly in the posterior aspect of the medial tibiofemoral compartment. This results in shearing forces that cause increasing damage to the articular cartilage. The patients have pain and swelling of their knees after this damage. The resulting instability is the most important factor leading to the progression of the arthritis and varus malalignment ^21^. The HTO can solve this problem. Many studies recommend that the combined operation (HTO and ligament reconstruction) is beneficial in the younger patients with pre-arthritis ^22, 23^, although some have recommended that it should be performed in two stages because of the potential morbidity ^24^. So some patients who continued to have instability of knee were operated in two stages in this study. T-locking plate (stainless steel 316L, 6hole) was used in all operations of HTO. Tomofix is a thick implant. While it is applied for fixation, skin and soft tissue is under tension and then complications (infection or hematoma) occur. Though T-locking plate is thinner than Tomofix its strength is optimal for this procedure. The thin T-locking plate does not cause skin tension, so this is advance on Tomofix. The cost of Tomofix is more than six fold compared to T-locking plate (stainless 316L, 6 holes).

In general, some complications of the HTO have been reported: infection (2.3–54.5%), hematoma (4.7%)^25, 26^, deep vein thrombosis (1.3–9.8%) ^27^, paresis of the peroneal nerve (2.0–16.0%) ^28, 29^, nonunion of the tibial osteotomy (0.7–4.4%) ^30, 31, 32^, vessel injury (0.4%) ^33^. Paresis of the peroneal nerve possibly occurred only with lateral closed wedge HTO. However, the patients in the current study had minimal invasive skin incision, so they did not have these complications. The only complication was in one case with an intra-articular fracture of the lateral tibial plateau that was observed on the postoperative radiograph, which delayed weight bearing by the patient; it had completely united as observed in the radiograph eight week later.

This study still has some limitations. Firstly, the number of cases is small, and the causes of varus deformity of the knees are different. Finally, although this short-term study shows good results, longer-term studies need to be conducted.The patients who had varus deformity caused by chronic ligament injuries must be particularly followed up after plate removal, and the ligaments reconstructed.

**Table I: Patient Demographics and Study Details:**
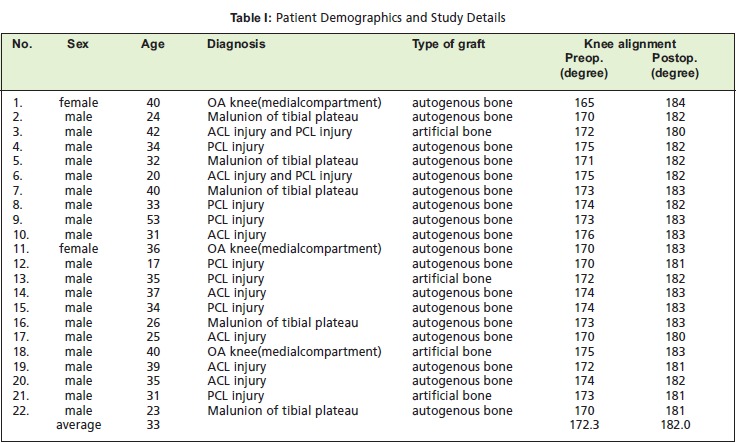


**Fig. 1a: The weight-bearing line (white line) was defined as the line connecting the hip and ankle centers. Varus and valgus angulations of the knee were measured by - the angle between the femoral and tibial mechanical axes (white dash line). Overcorrection of the new mechanical axis (white arrow line) according to - Fujisawa-:**
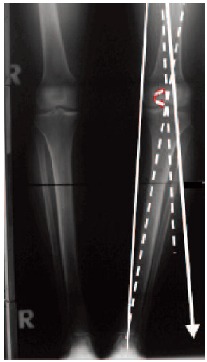


**Fig. 2: Skin incision (large black line) 5 cm is distal the joint line. The distal locking head screws were inserted through a small incision (small black line).**
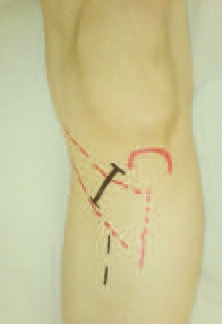


**Fig. 3: Clinical outcome evaluated by Lysholm score indicating that further subjective improvement was found. Statistical significance- of the difference - is shown (P <0.05).**
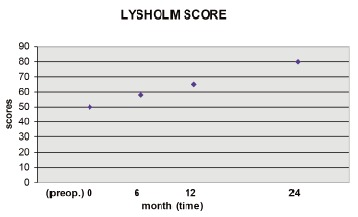


**Fig. 4: Pain evaluated using the visual analogue scale (VAS) from 0 to 10 (0=no pain, 10=severe pain). Significant pain reduction through follow up examinations was recorded (p<0.05).**
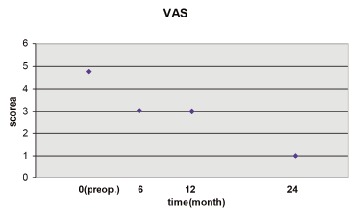


**Fig. 5: Intraoperative complications, an intra-articular fracture of the lateral tibial plateau was observed on a postoperative radiograph (Picture A), which delayed weight bearing. Ten week later, fracture had complete bony union and widening medial compartment on radiograph (Picture B). Postoperative radiography of a left knee 12 months postoperatively. Bony consolidation of the osteotomy gap with remodeling is visible (Picture C).**
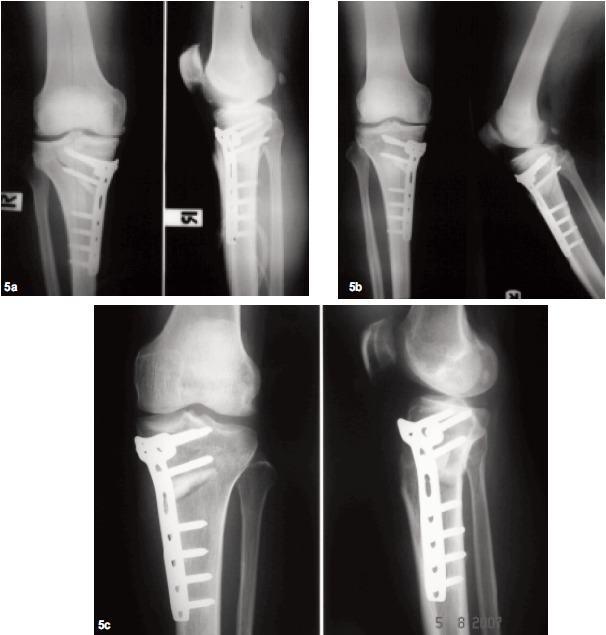


## Conclusion

Medial opening wedge osteotomy with T-locking plate (stainless steel 316L, 6 holes) is safe and an efficient procedure for correcting varus deformity of the knee.
